# Effect of step-synchronized vibration stimulation of soles on gait in Parkinson's disease: a pilot study

**DOI:** 10.1186/1743-0003-3-9

**Published:** 2006-05-04

**Authors:** Peter Novak, Vera Novak

**Affiliations:** 1 Department of Neurology, Boston University School of Medicine; 715 Albany Street, C315, Boston, MA 02118, USA; 2 Division of Gerontology^2^, Beth Israel Deaconess Medical Center, Harvard Medical School, Boston, MA, USA

## Abstract

**Background:**

Previous studies have suggested that impaired proprioceptive processing in the striatum may contribute to abnormal gait in Parkinson's disease (PD).

**Methods:**

This pilot study assessed the effects of enhanced proprioceptive feedback using step-synchronized vibration stimulation of the soles (S-VS) on gait in PD. S-VS was used in 8 PD subjects (3 women and 5 men, age range 44–79 years, on medication) and 8 age-matched healthy subjects (5 women and 3 men). PD subjects had mild or moderate gait impairment associated with abnormal balance, but they did not have gait freezing. Three vibratory devices (VDs) were embedded in elastic insoles (one below the heel and two below the forefoot areas) inserted into the shoes. Each VD operates independently and has a pressure switch that activates the underlying vibratory actuator. The VD delivered the 70-Hz suprathreshold vibration pulse upon touch by the heel or forefoot, and the vibration pulse was deactivated upon respective push-offs. Six-minute hallway walking was studied with and without S-VS. Gait characteristics were measured using the force-sensitive foot switches. The primary outcome was the stride variability expressed as a coefficient of variation (CV), a measure of gait steadiness. Secondary outcome measures were walking distance and speed, stride length and duration, cadence, stance, swing and double support duration, and respective CVs (if applicable).

**Results:**

The walking speed (p < 0.04) and the CV of the stride interval (p < 0.02) differed between the groups and S-VS conditions. In the PD group, S-VS decreased stride variability (p < 0.002), increased walking speed (p < 0.0001), stride duration (p < 0.01), stride length (p < 0.0002), and cadence (p < 0.03). In the control group, S-VS decreased stride variability (p < 0.006) and increased gait speed (p < 0.03), but other locomotion parameters were not significantly altered.

**Conclusion:**

Augmented sensory feedback improves parkinsonian gait steadiness in the short-term setting. Because the suprathreshold stimulation prevented blinding of subjects, the learning effect and increased attention can be a confounding factor underlying results. Long-term studies are needed to establish the clinical value of the S-VS.

## Background

Parkinson's disease (PD) is caused by a dopamine deficiency in the basal ganglia that results in characteristic motor abnormalities including postural instability and gait impairment. Short shuffling steps, slow walking speed, and increased stride variability characterize abnormal gait in PD. Although PD is primarily a motor disease, accumulating evidence suggests that abnormal proprioception and kinesthesia contribute to the parkinsonian gait. PD patients have reduced sensation on the plantar feet [1] and impaired joint position sense [2], movement perception [3], and movement accuracy [4-6]. It has been proposed that an inadequate integration of sensory inputs at the striatum and a defective proprioceptive feedback underlie abnormal motor control movement in PD [6,7].

Sensory feedback is necessary for postural adjustments and facilitates control of compensatory stepping reactions evoked by postural perturbation [8-10]. Cutaneous, joint, and muscular mechanoreceptors provide the necessary proprioceptive inputs [11]. Mechanical stimulation of foot mechanoreceptors can be used to perturb the proprioceptive feedback and to assess its role in generation of parkinsonian gait. The foot pressure activates the plantar mechanoreceptors that mediate postural adjustment during the stance phases of the step [10]. Several studies explored the effects of mechanical stimulation upon static balance as a mean for proprioceptive feedback modulation. Subsensory mechanical noise applied to the soles has improved the quiet-standing balance in healthy controls [12] and in patients with diabetes and stroke [13]. This effect was attributed to enhanced proprioceptive feedback. The effect of the suprathreshold stimulation is complex and depends on the frequency, amplitude, and location of the stimulation [14,15]. For example, during standing, the vibratory stimulation of the forefoot zones induces early electromyographic responses in the soleus muscle (mean latency 119 ms), followed by small forward center of pressure (CoP) displacement (mean latency 251 ms) and backward body tilt (mean latency 434 ms). Vibratory stimulation of rear foot zones has a similar effect but with an opposite direction of the body tilt. Simultaneous activation of both forefoot and rear foot zones has no effect on body tilt but does cause CoP oscillations. These results imply that characteristic postural responses may be specific to the localization and character of a stimulus.

We hypothesized that a vibration stimulation of foot mechanoreceptors synchronized with the step improves gait in PD. In this study the step-synchronized vibration stimulation was used to enhance the proprioceptive input during walking in healthy and PD subjects. The vibratory stimulus was delivered to the soles during the stance phase of the step, but not during the swing phase. Preliminary results were previously published as an abstract [16].

## Materials and methods

Eight subjects with a clinical diagnosis of idiopathic PD participated in the study. Clinical and demographic characteristics of the PD subjects are summarized in Table 1. Inclusion criteria for PD subjects were history of bradykinesia, rigidity, resting tremor, abnormal gait, asymmetric onset of symptoms, and good response to dopaminergic medication (if applicable) consistent with UK Brain Bank criteria [17]. Eight healthy subjects (5 women and 3 men, mean age 58.9 ± 12.3, range 45–75 years, mean weight 74.8 ± 6.4, range 67–84 kg, mean height 169.5 ± 8.5, range 157–185 cm) were age-matched with the PD group. Criteria for abnormal gait were mild to moderate difficulties while on medication that correspond to subscore 1–2 on the Unified Parkinson Disease Rating Scale (UPDRS), subscale II (Activities of daily living, walking subscore item 15 and item 29) during *on *state. Postural stability was evaluated using Motor Examination scale (subscale III, item 30). Subjects with moderate gait impairment (answer 2 in question 15) were eligible if they required no assistance with walking. An additional inclusion criterion was that subjects be able to walk for 6 minutes without interruption. Exclusion criteria were history of peripheral polyneuropathy, walking impairment due to arthritis, pain, muscle weakness, or cardiovascular or lung disorder. All subjects had a thorough neurological evaluation.

**Table 1:** Demographic and clinical characteristics of subjects with Parkinson's disease

**PD No.**	**Sex**	**Age (yrs)**	**Height (cm)**	**Weight (kg)**	**PD (yrs)**	**PD Stage**	**Unified Parkinson Disease Rating Scale**					**LEDD**
								
							**Total**	**Motor**	**Walk**	**Gait**	**PS**	
**1**	M	63	180	86	13	2.5	18.5	10.5	1	1	1	1080
**2**	F	45	163	57	3	2.5	23	18	1	1	1	600
**3**	F	59	162	61	7	2.5	47	27	2	1	1	800
**4**	M	79	173	72	3	2.5	32	17	1	1	1	500
**5**	M	72	182	81	10	2.5	32	22	2	1	1	1650
**6**	M	44	170	86	2	2	32	18	1	1	1	150
**7**	F	70	167.5	59	6	2.5	26	16	1	1	1	300
**8**	M	59	172	73	4	2.5	27	18	1	1	1	75
**Mean**		61.4	171.2	71.9	6.0	2.4	29.7	18.3	1.25	1	1	725.7
**SD**		12.4	7.2	11.9	3.9	0.2	8.5	4.7	0.5	0	0	510.1

No. = subject number, Stage = Hoehn and Yahr Disability Scale, walk = item 15 and gait = item 29 (Activities of daily living, Subscale II), PS (postural stability) = item 30, (Motor Examination, Subscale III), evaluated during the *on *state

LEDD = the levodopa equivalent daily dose

Subjects were included if they were able to walk for 6 minutes at self-paced speed without interruption. Subjects were excluded if they had medical history of peripheral polyneuropathy, hypertension, stroke, CNS or gait disorder, or diabetes or if they used walking aids. All healthy subjects had normal gait. The Institutional Review Board of Boston University approved the study, and all subjects signed a written informed consent.

### Vibratory Device

**Figure 1:**
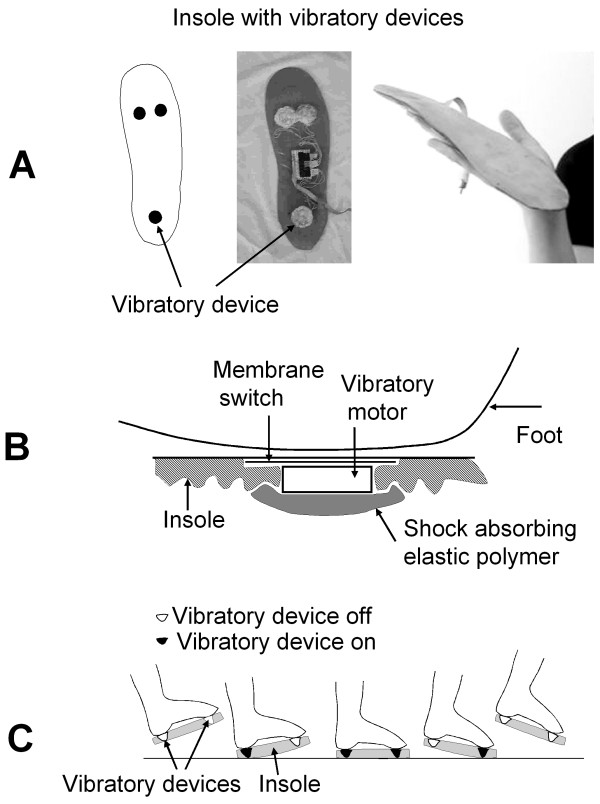
The insole with the vibratory device (A) and schematic diagram that shows integration of the vibratory device within the insole (B) and sequence of the vibratory stimulation during step phases (C). Vibratory device consists of a vibration disk motor (diameter 18 mm) and a membrane switch attached to the top of the motor, with a resulting thickness of ~5 mm and weight of ~5 grams.

A wearable, battery-operated vibratory device (VD) delivers a vibration stimulus to the soles that is synchronized with the step (Figure 1). Three VDs were embedded in each insole: one below the heel, and two below the forefoot. The VD senses pressure on the sole and delivers the vibration stimulus upon touch of the heel or forefoot. The vibration stimulation is turned off during the swing phase of gait. The VD delivers suprathreshold stimulation that is perceived as a light vibration at the soles. Vibration intensity is similar to that of portable devices such as cell phones and beepers, operating in the vibration mode. VD was mounted on shoe insoles inserted into the shoes. The VD utilizes the miniature vibrating disk motor Optec 2890W11 (OPTEC Co., Ltd., Japan) vibrating at the frequency 70 Hz and operating at 1.3 Volts. The vibratory device consists of a vibration disk motor (diameter 18 mm) and a membrane switch glued on top of it. The resulting thickness is ~5.0 mm, weight is ~5 grams, and vibration range is 0.1 – 0.2 mm.

The foot sensor that provides feedback to the VD is based on an industrial membrane switch that turns on with the force 350 g (Nelson Nameplate, Inc., Los Angeles, CA). The foot sensor is attached on top of the vibration motor enclosure. The VD (e.g., vibratory motor + membrane switch) is embedded in the elastic insoles (Dr. Scholl's massaging gel insoles^®^, Shering-Plough, Kenilworth, NJ). The VD is isolated from the shoe by shock-absorbing elastic silicon polymer. Each VD is activated independently, i.e., the heel switch controls the heel vibratory motor such that heel stimulation starts with heel touch and stops upon heel off. The forefoot switches control the underlying forefoot actuators that turn on upon forefoot touch and turn off upon toes lifting. This means that different parts of the sole are stimulated at different sub-phases of the step.

### Study Protocol

The walking trials were done in the *on medication *state in PD subjects. The insoles with VDs were inserted into the subject's shoes. Subjects walked for 6 minutes (6-minute walk test, [14]) at a self-paced speed in the hallway (length 73 m, width 1.7 m) with the VD turned *off*, and then they had a 5-minute sitting rest. Next, subjects walked for 6 minutes with the VD turned *on*. Subjects were not informed about the outcome measures. They were asked to walk comfortably at their normal walking speed, and they were specifically instructed not to walk faster or slower than their most comfortable level. All PD subjects were well familiar with the test place, where they had walked several times before. Any encouragement throughout the walking trials was avoided since it might affect the gait profile [18]. To reduce expectation bias, subjects were allowed to walk for about 1 minute with the device *on *and *off *before the gait recordings. An investigator followed the study subjects during walking trials as a safety measure, and he also measured the gait distance with a "Meter-Man" distance-measuring wheel (Winnebago, MN).

### Data Acquisition and Processing

Gait signals were recorded using the Gait Logger (JAS Research. Inc., Boston, MA) connected to the foot switches with 4 force sensors on each foot (B&L Engineering, Inc., Tustin, CA). Gait signals were sampled at 200 Hz per switch using a 16-bit analog/digital converter and recorded on the portable microcontroller-based storage device. The raw data were processed off-line using the software written in Matlab^® ^6.1 (The MathWorks, Inc., Natick, MA). Turns were excluded from statistical analysis since gait variability can be affected by a particular turning pattern (e.g., turning in a small circle versus sudden 180 degree rotation). Stride, stance, swing, and double support duration were computed in each gait cycle (in milliseconds and as a percentage of the gait cycle) and averaged over each walking trial. The primary outcome measure was the stride variability expressed as the coefficient of variation (CV) of the stride interval, which is a measure of gait steadiness [19]. Secondary outcome measures were the following gait parameters: walking distance and speed, stride length and duration, cadence, stance, swing duration, double support, and their respective CVs (if applicable). Gait parameters were averaged between the right and left legs for statistical analysis.

Statistical analysis was performed using statistical software JPM 5.1 (SAS Institute, Cary, NC). The effects of vibratory stimulation between the conditions (S-VS *on *vs. S-VS *off*) and groups were compared using MANOVA adjusting for age, sex, and height. Paired t-test was used to compare effects of vibratory stimulation within each group.

## Results

Demographic characteristics (age, height, and weight) did not differ between the PD and the control groups.

### Walking without step-synchronized vibration stimulation

Six-minute walking trials included the straight segments and typically 4–6 turns at 180 degrees. There were no gait-freezing episodes or falls. *PD subjects *had significantly slower walking speed and higher CV of the stride interval, stance, and doubles support than did control subjects (Table 2). Other locomotion parameters were not significantly different between the groups.

**Table 2:** Gait characteristics in the Parkinson's disease and control groups during 6-minute walking with and without step-synchronized vibration stimulation

**Locomotion Parameters**	**Parkinson's Disease Group**			**Control Group**			Manova
	
	**S-VS OFF**	**S-VS ON**	p_G_	**S-VS OFF**	**S-VS ON**	p_G_	p
Walking distance (m)	368 ± 73.4	402.7 ± 72.6	0.0001	453.1 ± 53.2	476.1 ± 61.6	0.03	0.02
Velocity (m/s)	1.02 ± 0.2	1.11 ± 0.2	0.0001	1.25 ± 0.2	1.32 ± 0.17	0.03	0.04
Cadence (steps/min)	104.9 ± 8.9	109.2 ± 10.2	0.03	110.9 ± 4.9	112 ± 5.7	0.11	0.21
Stride duration (ms)	1149.6 ± 90.9	1107 ± 100.9	0.01	1112.9 ± 99.0	1103.2 ± 105.4	0.11	0.25
Stride length (m)	1.17 ± 0.24	1.24 ± 0.3	0.0002	1.4 ± 0.16	1.37 ± 0.19	0.06	0.06
Stride CV (%)	5.36 ± 3.1	4.4 ± 2.7	0.002	2.8 ± 0.4	2.3 ± 0.5	0.006	0.02
Stance duration (ms)	730.8 ± 79.7	679.3 ± 90.2	0.04	653.8 ± 66.19	654.95 ± 69.9	0.8	0.04
Stance CV (%)	1.99 ± 1.0	1.6 ± 0.8	0.1	1.29 ± 0.63	0.99 ± 0.30	0.15	0.11
Swing duration (ms)	418.8 ± 54.8	427.7 ± 64.6	0.75	446.6 ± 83.4	435.8 ± 85.8	0.09	0.37
Swing CV (%)	1.86 ± 1.04	1.6 ± 0.8	0.33	0.95 ± 0.4	0.88 ± 0.45	0.09	0.12
Double support duration (ms)	156.0 ± 51.1	134.6 ± 42.8	0.37	115.6 ± 25.7	112.1 ± 45.7	0.26	0.08
Double support CV (%)	2.78 ± 1.6	2.77 ± 1.7	0.06	0.72 ± 0.25	0.97 ± 0.87	0.43	0.05

Mean ± SD

SV OFF – walking without step-synchronized vibration stimulation, S-VS ON walking with step synchronized vibration stimulation

p = Manova comparisons between the groups and S-VS conditions, p_G _= within group comparisons using paired t-test

CV – coefficient of variation

### Step-synchronized vibration stimulation

The vibratory device was well tolerated; none of the subjects experienced gait freezing or falls. The most common experience was an increased awareness of the foot placement on the floor. The walking speed (p < 0.04) and the CV of the stride interval (p < 0.02) differed between the groups and between the S-VS *on *and S-VS *off *conditions (Table 2). The walking speed increased and the CV of the stride interval decreased during the S-VS *on walking *as compared to the S-VS off walking. Other locomotor parameters (cadence, stride, and swing duration) did not differ significantly either between groups or between S-VS conditions. There were no significant differences between the left and right legs in the stride interval and its corresponding CV.

#### Parkinson's disease group

**Figure 2:**
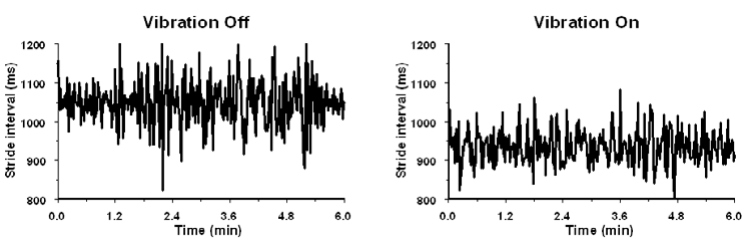
Stride intervals of the left leg from a 45-year-old Parkinson's disease subject obtained during 6-minute walking without (left) and with (right) the vibratory stimulation of soles. Vibratory stimulation reduced the coefficient of variation of stride interval from baseline value 11.49% (SD = 62.0 ms) to 9.43% (SD = 44.2 ms).

Figure 2 shows an example of the stride intervals measured during walking with and without the S-VS in a PD subject (subject no. 2 in Table 1). Walking with the S-VS significantly increased the walking speed (p < 0.0001), cadence (p = 0.03), stride duration (p = 0.01), and stride length (p = 0.0002). The CV of the stride intervals (p = 0.0002) and the stance duration (p = 0.04) decreased during the S-VS walking. The stance percentage of the step, double support duration, double support percentage of the step, and coefficient of variation of the double support were not affected. Two PD subjects with histories of falls (subject no. 2 and no. 3 in Table 1) had the highest baseline coefficient of variation of the stride. In these subjects the S-VS improved the CVs of their stride intervals by 20.9% and 32%, respectively.

#### Control group

The walking speed increased (p = 0.03) and the CV of the stride interval decreased (p = 0.006) during the S-VS walking. Other locomotion parameters were not significantly altered by the S-VS.

## Discussion

In this study, vibration stimulation of the foot soles synchronized with the step increased the walking speed and improved the stride variability in PD subjects. In addition, vibration stimulation prolonged the stride interval and the stride length. Stride variability also decreased in the control group. Stride variability, which is an important measure of motor performance and gait unsteadiness, is increased in subjects with a history of falls [19-23] and is an independent predictor of falling [19]. The step-synchronized vibration may stabilize gait in PD patients by reducing the stride variability.

The vibration stimulus was suprathreshold, a situation that prevented blinding of the study participants. The increased awareness of foot placement may affect gait characteristics, as suggested by the effects of attention strategies [24]. The subjects were instructed to walk at a comfortable speed without any reference to gait attention to minimize the unspecific effects of gait awareness. Therefore, it is not likely that increased attention may account for all S-VS effects.

In our study, subjects were walking at a comfortable pace, without any encouragement or instructions might affect their walking speed. The mean walking distance increased by 9.4% in the PD group and by 5.2% in the control group during the S-VS walking. The 6-minute walking test (6MWT) is believed to reflect activities of daily living, but there might be a placebo response and training effect among repetitive walking trials [18,25]. For example, one study found an 8% increase in walking distance on the second trial in healthy elderly (2.5-hour break between the trials)[26]; another found a 3% increase in patients with fibromyalgia (1-day break between the trials) [27]; and a third found a 4.8% increase in patients with heart failure (30 minute break between the trials) [28]. Direct comparisons of these repetitive walking trials are problematic, as the methodology differed among them. For example, subjects were asked to "walk a pace that was brisk but comfortable" without encouragement [27], to "cover as much distance as possible until exhausted" without encouragement [28], or to walk at their own maximal pace with encouragement every 30 seconds [26]. Furthermore, the stride variability in repetitive 6MWT was not measured, and the effects of repetitive 6MWT trials on walking distance in PD patients are unknown. Our study differs from the above trials not only in terms of the patient population, the much shorter inter-trial breaks, and the lack of encouragement, but also in the fact that we took several measures to minimize the learning effect. These measures included walking in a familiar environment (PD group), using specific instructions to discourage subjects to walk faster (or slower) than at their most comfortable speed, and having each subject walk for as long as 1 minute with the vibratory devices turned on and off before the actual walking recordings. Nevertheless, as the placebo and learning effect cannot be completely excluded, only a long-term study in a larger patient population can provide robust measures of the effects of S-VS walking.

The effect of S-VS is likely to be related to enhanced proprioceptive feedback, even upon considering other possible confounders. Locomotor patterns are regulated through the feedback loops among the proprioceptive receptors and central motor pattern generators. Sensory feedback is necessary for gait stability in that it provides inputs to the central pattern generators that can instantly adapt to external perturbations and correct programming errors in intended movement direction, force, and execution [29-31]. The vibration device in our study operated in a simple closed loop mode wherein the enhanced feedback was synchronized with the distribution of plantar pressures during the gait cycle phase. Therefore, the synchronization of vibration stimulation with the gait phase may improve timing and variability of the gait cycle by enhanced recruitment of sensorimotor pathways including spinal circuitry and basal ganglia. Supporting this notion are functional magnetic resonance imaging studies that have demonstrated activation of distinct brain structures when vibration stimulus was used [32,33]. Stimulation of the fingertips activated the contralateral primary somatosensory cortex, bilateral secondary somatosensory cortex, the precentral gyrus, the posterior insula, the posterior parietal region, and the posterior cingulate [33]. Positron emission tomography studies showed that stimulation of the metacarpal joints activated ipsilateral sensory cortical areas and contralateral basal ganglia [32].

Results of this study, however, may be not applied to the whole PD population given our small sample and selection of patients. The PD subjects had mild to moderate gait impairment that was predominantly associated with abnormal balance. None of the subjects had the gait freezing episodes commonly seen in more advanced disease. Gait freezing is a poorly understood phenomenon that may be due to pathophysiological mechanisms different from those causing abnormal balance [34].

## Conclusion

This study indicates that the step-synchronized vibration stimulation of the soles improves gait steadiness in Parkinson's disease patients with predominant balance impairment. The suprathreshold stimulation improved gait, presumably by enhancing the sensory feedback. Previous reports showing impaired proprioception support this notion. In this short-term non-blinded design, possible placebo and learning effects cannot be completely excluded. Long-term studies are needed to establish a clinical value of the S-VS.

## Abbreviations

PD Parkinson's disease

S-VS step-synchronized vibration stimulation

VD vibratory device

CV coefficient of variation

CoP center of pressure

UPDRS Unified Parkinson Disease Rating Scale

6MWT 6-minute walking test

LEDD levodopa equivalent scale

## Competing interests

A patent for the device described in this study has been filed with the US Patent and Trademark Office. The patent is property of Boston Medical Center Corporation.

## Authors' contributions

P.N. designed the device, the study and conducted the experiments, data analysis, interpretation, and manuscript preparation.

V.N. contributed to study design and participated in data analysis, interpretation, and manuscript preparation.
